# Molecular analyses of *Fusarium* isolates recovered from a cluster of invasive mold infections in a Brazilian hospital

**DOI:** 10.1186/1471-2334-13-49

**Published:** 2013-01-30

**Authors:** Christina M Scheel, Steven F Hurst, Gloria Barreiros, Tiyomi Akiti, Marcio Nucci, S Arunmozhi Balajee

**Affiliations:** 1 Mycotic Diseases Branch, Centers for Disease Control and Prevention, Atlanta, GA, USA; 2 University Hospital, Universidade Federal do Rio de Janeiro, Rio de Janeiro, Brazil; 3 Center for Global Health, Centers for Disease Control and Prevention, Atlanta, GA, USA

**Keywords:** *Fusarium*, Invasive fusariosis, Molecular epidemiology, Multi-locus sequence typing, Invasive fungal infection, Bone marrow transplant, Hospital-associated infection, Community acquired disease, Brazil

## Abstract

**Background:**

Invasive fusariosis (IF) is a rare but often fatal fungal infection in immunosuppressed patients. In 2007, cases of IF above the expected epidemiologic baseline were detected in the hematology ward of a hospital in Rio de Janeiro, Brazil. Possible sources of infection were investigated by performing environmental sampling and patient isolate collection, followed by molecular typing. Isolates from dermatology patients with superficial fusariosis were included in the study for comparison to molecular types found in the community.

**Methods:**

Environmental sampling focused on water-related sources in and around the hematology ward. Initially, we characterized 166 clinical and environmental isolates using the *Fusarium* translation elongation factor 1α (*EF-1α*) genetic locus. Isolates included 68 collected from water-related sources in the hospital environment, 55 from 18 hematology patients, and 43 from the skin/nails of 40 outpatients seen at the hospital dermatology clinic. Multi-locus sequence typing was performed on *Fusarium solani* species complex (FSSC) species 1 and 2 isolates to investigate their relatedness further.

**Results:**

Most of the hematology samples were FSSC species 2, with species type FSSC 2-d the most commonly isolated from these patients. Most of the outpatient dermatology samples were also FSSC 2, with type 2-d again predominating. In contrast, environmental isolates from water sources were mostly *Fusarium oxysporum* species complex (FOSC) and those from air samples mostly *Fusarium incarnatum-equiseti* species complex (FIESC). A third of the environmental samples were FSSC, with species types FSSC 1-a and FSSC 1-b predominating.

**Conclusions:**

*Fusarium* isolate species types from hematology patient infections were highly similar to those recovered from dermatology patients in the community. Four species types (FSSC 1-a, 1-b, 2-d and 2-f) were shared between hematology patients and the environment. Limitations in environmental sampling do not allow for nosocomial sources of infection to be ruled out. Future studies will focus on environmental factors that may have influenced the prevalence of FSSC fusariosis in this hematology ward.

## Background

Over the last two decades, invasive mold infections have become increasingly common among severely ill hospital patients worldwide [1,2]. Fusariosis may manifest as superficial or skin, nail and eye infections in healthy persons, whereas severely immunocompromised patients can develop invasive disease [2-5]. Invasive fusariosis (IF) is a devastating fungal infection with high mortality rates in patients with hematological malignancies and in hematopoietic stem cell transplant recipients [3].

Since 2005, an increase in cases of fusariosis was observed in a hospital in Rio de Janeiro, Brazil, both among immunosuppressed patients in the bone marrow transplant unit (BMTU) and also among persons with superficial infections who were treated at the local dermatology clinic. In 2007, the number of cases of fusariosis in the BMTU above the expected epidemiologic baseline was recorded. Periodic environmental sampling in the hospital, focused on water and water-related sources within the BMTU, commenced in 2005 and was continued until 2009, in an attempt to identify possible environmental reservoirs of infection. Beginning in 2006, *Fusarium* isolates from hospital patients, hospital environmental samples, and dermatology clinic patients were archived for further molecular analysis. Here we report the results of comparative sequence typing performed on these archived isolates to assess the ecology of the hospital environment, determine the molecular genetic relatedness between patient and environmental samples, and to estimate the degree to which environmental *Fusarium* could be a reservoir for infection within the hospital.

## Methods

### Sampling and isolates

This study was conducted in the University Hospital, Federal University of Rio de Janeiro, Brazil. The University Hospital is a 13-floor tertiary care teaching hospital with ~480 beds. The BMTU is an isolated 18-bed ward located on the eighth floor of the building. Rooms in the BMTU are under positive pressure air flow, and 8 beds have high-efficiency particulate air (HEPA) filters. Sporadic cases of IF had been diagnosed in the BMTU at a rate of 1.47 cases per 1000 admissions between 2000 and 2006. In 2007, there was a sharp increase in the rate of fusariosis, reaching 16.78 cases per 1000 BMTU admissions. During this period, there was a concurrent increase of superficial fusariosis in outpatients seen at the dermatology clinic on the hospital’s second floor.

Fifty-five *Fusarium* isolates were recovered from 18 hematology patients (38 isolates from sterile site samples and 17 from non-sterile sites) in the BMTU as a part of standard medical care between December 2005 and September 2009. Forty-three *Fusarium* isolates were collected from skin and nail cultures of superficial lesions (onychomycosis and intertrigus) of 40 dermatology outpatients between June 2007 and October 2009 as part of standard medical care. After collection, all patient isolates were plated on Sabouraud dextrose agar (SDA), Mycosel, brain/heart infusion (BHI), and yeast extract agars and incubated at 25°C.

Hospital environmental sampling was performed periodically in the BMTU between June 2005 and Aug 2006 as part of an environmental surveillance program focused on air, water, and water-related surfaces. In September 2006 - October 2007, environmental sampling was performed in rooms of infected patients, with an emphasis on bathroom surfaces (shower heads, drains, and tile grout and sink surfaces and drains). Common areas of the BMTU were also swabbed as potential point sources of fungal contamination. Thereafter, routine environmental surveillance of the BMTU continued through July 2009. Environmental sampling of the outpatient dermatology clinic was not performed.

Surfaces were sampled using Culturette swabs (Becton Dickinson, Franklin Lakes, NJ, USA), inoculated onto SDA plates containing 50 mg/L chloramphenicol (SDA+C), and incubated at 25°C. Water (100 ml) was collected in sterile glass bottles and was filtered through 0.45 μm membranes (Millipore, Billerica, MA) which were placed on SAB plates and incubated at 25°C. Air was sampled from patient rooms in November 2006, August 2007, and July 2009 using a six-stage Anderson bioaerosol sampler (Anderson Instruments, Atlanta GA) with air impacting on an SDA plate for 30 min, for a total volume of 849 L per sample. Control air samples were also collected from other areas of the hospital, including the intensive care unit (2005, 2007 and 2009), pediatrics unit (2007) and the nephrology dialysis unit (2007).

*Fusarium* were isolated from 68 samples, ranging in time of collection from June 2005 to October 2009. Provisional identification of hematology patient *Fusarium* isolates were obtained in order to decide if more dramatic measures should be implemented in the BMTU. This identification was based on the micromorphology that is typical of this genus [6].

### Molecular identification and subtyping

All available *Fusarium* isolates collected from patients and the environment were sent to the Centers for Diseases Control and Prevention, Atlanta, GA, USA for molecular identification. At the CDC, isolates were sub-cultured on SDA+C with 50 mg/ml gentamycin agar, and incubated at 30°C for approximately 72 hours. Genomic DNA was extracted using the Qiagen DNeasy tissue kit (Qiagen, Valencia, CA) with slight modifications to the manufacturer’s instructions. Briefly, small sections of sub-cultured *Fusarium* (1 cm^2^) were cut from mycelial mats and placed in 5 ml polypropylene tubes containing 900 μl Qiagen ATL buffer and 60 U proteinase K. The mycelia in each tube were homogenized using the Omni TH Mixer (Omni Intl., Kennesaw, GA) at slow speed for 30 s and then high speed for 30 s, using a clean probe between each isolate. Homogenates were capped and incubated at 55°C for 1 h with frequent vortexing and then cooled to RT. RNase A (Sigma-Aldrich Corp., St. Louis, MO) was then added to a final concentration of 1 mg/ml and incubated for 5 min at RT, followed by the addition of 900 μl Qiagen buffer AL and vortexing. Homogenates were incubated at 70°C for 10 min, then transferred to 1.7 ml microcentrifuge tubes and centrifuged at 15,000 X g for 10 min. Clear supernatants (1 ml each) were transferred to clean microcentrifuge tubes and 500 μl ethanol (Sigma-Aldrich Corp.) was added. The suspensions were vortexed and transferred to Qiagen DNeasy columns, and manufacturer’s instructions were followed throughout the remainder of the procedure.

A region of the translation elongation factor-1α (*EF-1α*) gene was used to resolve all *Fusarium* isolates to the species complex level [7] [Genbank: HM852032-HM852059]. Multi-locus sequence typing (MLST) was performed on selected isolates. The *EF-1α* and two additional genetic loci, the internal transcribed spacer (ITS) including D1/D2 and large nuclear subunit (LSU) rRNA regions (*rRNA*) and a portion of the RNA polymerase II subunit (*RPB2*) gene, were amplified using primers described by O’Donnell *et al*. to determine species type (ST) [7] (Table 1). Additional primers were developed for this study to sequence the 5’ (F2F and F1R) and 3’ (40-2f and 40-r) of *RPB2* (Table 1). PCR was performed in 25 μl reactions containing 0.2 μM each forward and reverse primer, 2.5 mM MgCl_2,_ 0.2 mM each dNTP, and 0.625 U Taq DNA Polymerase (Roche Carolina Inc., Florence, SC) in a buffer of 10 mM Tris pH 8.3/50 mM KCl. Thermal cycling was performed in MicroAmp 96-well optical reaction plates using the GeneAmp PCR System 9700 (Applied Biosystems, Inc., Foster City, CA) for forty cycles as follows: 94°C for 5 min, 10 cycles of 94°C for 30 s, 60°C for 30 s (decreasing 1°C each cycle), 72°C for 30 s; then 30 cycles as above, but with the annealing temperature held at, 50°C for 30 s, and a final step of 72°C for 5 min. Amplicons were sized on 1.75% agarose and visualized with ethidium bromide under ultraviolet light. Amplicons were purified using Exo-SAP-IT (USB Corp., Cleveland, OH) according to manufacturer’s instructions, followed by sequencing with locus-specific primers using the BigDye Terminator v3.1 Cycle Sequencing Kit (Applied Biosystems Inc.) in a 3730 DNA Analyzer (Applied Biosystems, Inc.). Consensus sequences were generated from raw data using Sequencher 4.9 software (Gene Codes Corp., Ann Arbor, MI).

**Table 1:** **Primers used in multi-locus sequence typing of *Fusarium *isolates collected from patients and the environment**

**Locus**	**Gene product (size in bp)**	**Primer**	**Sequence 5’-3’**^**†**^	**PCR**	**Sequencing**
***EF-1α***	Translation elongation factor 1 alpha (716)	EF1	ATGGGTAAGGARGACAAGAC	x	
		EF2	GGARGTACCAGTSATCATG	x	
		EF3	GTAAGGAGGASAAGACTCACC		x
		EF22T	AGGAACCCTTACCGAGCTC		x
***rRNA***	Nuclear ribosomal rRNA (986)	ITS5	GGAAGTAAAAGTCGTAACAAGG	x	x
		ITS2	GCTGCGTTCTTCATCGATGC	x	x
		ITS3	GCATCGATGAAGAACGCAGC	x	x
		ITS4	TCCTCCGCTTATTGATATGC	x	x
		NL4	GGTCCGTGTTTCAAGACGG	x	x
***RPB2***	RNA Polymerase beta subunit (1738)	5f2	GGGGWGAYCAGAAGAAGGC	x	
		7cr	CCCATRGCTTGYTTRCCCAT	x	
		7cf	ATGGGYAARCAAGCYATGGG	x	
		11ar	GCRTGGATCTTRTCRTCSACC	x	
		F2F	AAAGCTCGCCAAGCCCCGTC		x
		F1R	GTCCTCTGGTGGCTCCGCCT		x
		40-2f	CAAAAACCTCTGGCGACAAC		x
		40-r	AGCTTGCGTCCAGTATGACC		x

^**†**^ Letter designations for degenerate bases: R, A or G; S, C or G; W, A or T; Y, C or T.

### Comparative sequence and phylogenetic analysis

*Fusarium* species complexes and STs were identified using the FUSARIUM ID database BLAST search feature [8] [Genbank: JX156437-JX15644, Genbank: JX282603-JX282609]. Consensus sequences were aligned using Clustal W and cleaned within the BioEdit Sequence Alignment Editor [9,10].

Neighbor-joining (NJ) trees were generated using Kimura’s two-parameter model and bootstrapped using 1000 random replicates with the Phylip 3.69 software package [11]. Resultant trees were then analyzed to generate a consensus NJ tree that was annotated using FigTree v.1.3.1 software [12]. Maximum Likelihood (MJ) and Maximum Parsimony (MP) algorithms were used to generate alternate trees for comparison to the NJ tree to ensure its validity.

## Results

### Molecular typing of EF-1α

**Figure 1:**
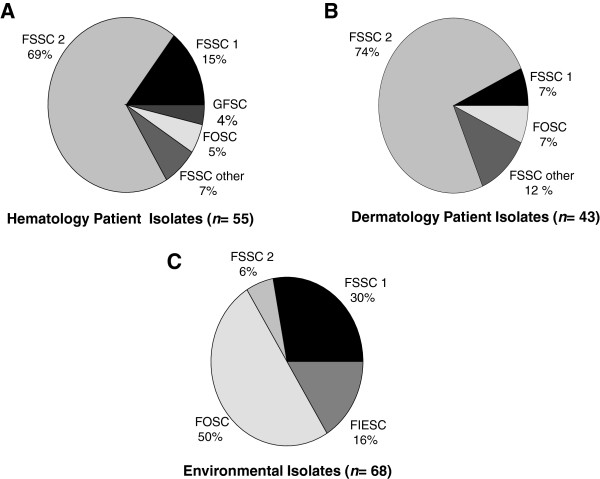
**Molecular typing of *Fusarium *isolates (*n *= 168) collected from patients and the environment.** Molecular typing to species complexes and species was accomplished by amplification and genetic sequencing of the translation elongation factor 1α (*EF-1α*) gene. Identities of study isolates were resolved by comparison of *EF-1α* molecular sequences to sequences of known *Fusarium* species archived in a in a curated database, FUSARIUM ID [8]. FSSC: *Fusarium solani* species complex; FOSC: *Fusarium oxysporum* species complex; FIESC: Fusarium *incarnatum-equiseti* species complex; GFSC: *Gibberella fujikuroi* species complex.

A total of 166 *Fusarium* isolates were available for analysis. These included 98 clinical isolates (55 from 18 hematological patients, 43 from 40 dermatology patients) and 68 isolates from environmental samples, including 14 air samples, 44 swab samples, and 10 water samples. Comparative *EF-1α* sequence analysis using the FUSARIUM ID database BLAST feature allowed for identification of most study isolates to the species level, with sequence identities ranging from 94.1% to 100% (mean = 99.9, median = 100%) when compared with database isolates. Frequencies of different species complexes/species isolated from patients and the environment are shown in Figure 1. Isolates from BMTU patients and dermatology patients showed a similar distribution. The majority of isolates (68%, 113/166) were identified as *Fusarium solani* species complex (FSSC). Among clinical isolates, FSSC species 2 (FSSC 2) predominated, and comprised 69% (38/55) of those collected from hematology patients, and 74% (32/43) of those collected from dermatology patients (Figure 1). *Fusarium oxysporum* species complex (FOSC) isolates were found in much greater numbers in the hospital environment (50%, 34/68) than in those collected from hematology (5%, 3/55) and dermatology (7%, 3/43) patients. *Gibberella fujikuroi* (GFSC) were isolated exclusively from hematology patients and *Fusarium incarnatum-equiseti* (FIESC) exclusively from environmental sources. FSSC 1 and FSSC 2 clinical and environmental isolates shared between 99.7 -100% *EF-1α* identity with FUSARIUM ID isolates, and were chosen for MLST due to their predominance in patients.

### Multi-locus sequence typing of FSSC and phylogenetic analysis

**Figure 2:**
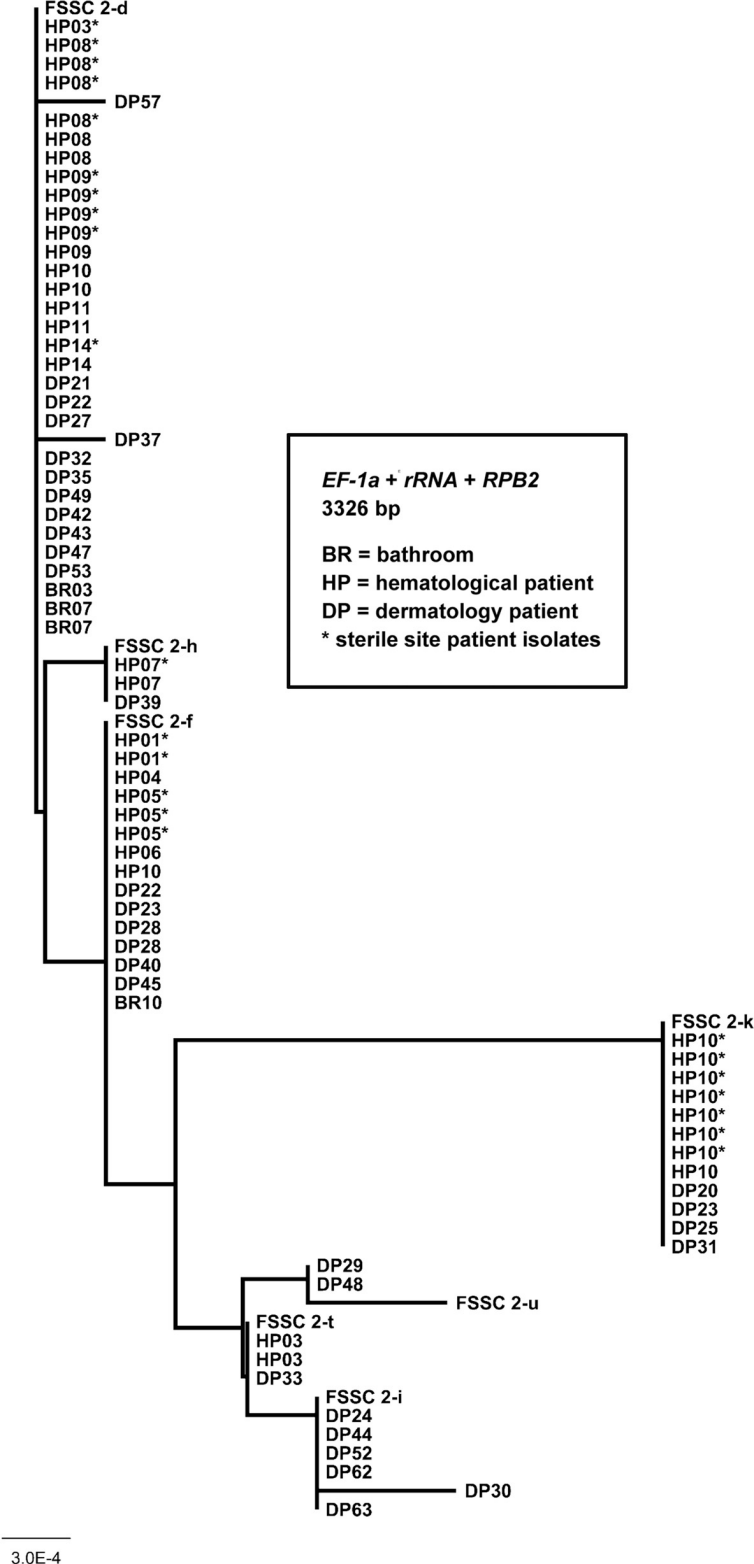
**Neighbor-joining phylogeny of *Fusarium solani *species complex (FSSC) species 2 isolates (*n *= 74).** Bootstrap values were 100% between individual taxa (1000 iterations, data not shown). Included are previously described FSSC species 2 voucher sequences (labeled FSSC 2-d, FSSC 2-f, FSSC 2-h, FSSC 2-I, FSSC 2-k, and FSSC 2-u) that were retrieved from the FUSARIUM ID database [8].

Species type identifications of 104 FSSC isolates (FSSC 1 = 30, FSSC 2 = 74) by MLST and *EF-1α* species complex/ species identification (42 isolates) are shown in Table 2. The cladogram in Figure 2 represents all FSSC 2 isolates described in this study. No cladogram was prepared for FSSC 1-a and 1-b isolates, since these STs differ by only one base pair.

**Table 2:** **Speciation of clinical and environmental *Fusarium *isolates collected in a Brazilain hospital**

**Species ID (*EF-1α *Identity match%)**	**Isolate number**	**Collection date**	**Sample type**	**Isolate source**
FSSC^†^ (99.3)	B8586	12/10/2005	Clinical	HP01, blood
FSSC^†^ (100)	B8659	10/16/2007	Clinical	DP33, skin scraping
FSSC^†^ (99.1)	B8684	7/13/2009	Clinical	DP58, nail scraping
FSSC^†^ (99.6)	B8685	7/31/2009	Clinical	DP60, skin scraping
FSSC 1-a	B8648	7/16/2007	Clinical	DP26, nail scraping
FSSC 1-a	B8593	7/19/2006	Clinical	HP03, lesion swab
FSSC 1-a	B8710	7/31/2007	Environmental	BR06, shower swab
FSSC 1-a	B8711	7/31/2007	Environmental	BR06, shower swab
FSSC 1-a	B8712	7/31/2007	Environmental	BR06, wall swab
FSSC 1-a	B8714	7/31/2007	Environmental	BR06, wall swab
FSSC 1-a	B8715	7/31/2007	Environmental	BR06, wall swab
FSSC 1-a	B8716	7/31/2007	Environmental	BR06, wall swab
FSSC 1-a	B8720	8/14/2007	Environmental	BR07, shower swab
FSSC 1-a	B8721	8/14/2007	Environmental	BR07, shower swab
FSSC 1-a	B8690	9/21/2006	Environmental	BR10, wall swab
FSSC 1-a	B8694	10/5/2006	Environmental	BR11, floor swab
FSSC 1-a	B8741	10/25/2007	Environmental	BR12, shower swab
FSSC 1-a	B8696	11/1/2006	Environmental	BR03, air
FSSC 1-b	B8589	4/17/2006	Clinical	HP02, blood
FSSC 1-b	B8590	4/17/2006	Clinical	HP02, blood
FSSC 1-b	B8591	4/20/2006	Clinical	HP02, skin biopsy
FSSC 1-b	B8700	11/1/2006	Environmental	BR03, floor swab
FSSC 1-b	B8717	7/31/2007	Environmental	BR06, wall swab
FSSC 1-b	B8728	8/31/2007	Environmental	BR03, sink swab
FSSC 1-b	B8744	10/26/2007	Environmental	BR01, water
FSSC 1-b	B8745	10/26/2007	Environmental	BR01, water
FSSC 1-b	B8662	10/31/2007	Clinical	DP36, nail scraping
FSSC 1-b	B8681	5/20/2009	Clinical	DP55, skin scraping
FSSC 1-b	B8757	7/6/2009	Environmental	BR03, shower swab
FSSC 1-b	B8760	7/6/2009	Environmental	BR03, air
FSSC 1-b	B8637	7/28/2009	Clinical	HP17, biopsy
FSSC 1-b	B8638	9/4/2009	Clinical	HP18, blood
FSSC 1-b	B8639	9/4/2009	Clinical	HP18, blood
FSSC 1-b	B8640	9/4/2009	Clinical	HP18, blood
FSSC 2-d	B8592	7/12/2006	Clinical	HP03, blood
FSSC 2-d	B8697	11/1/2006	Environmental	BR03, air
FSSC 2-d	B8601	4/10/2007	Clinical	HP07, skin biopsy
FSSC 2-d	B8602	4/10/2007	Clinical	HP07, vesicle
FSSC 2-d	B8643	6/14/2007	Clinical	DP21, nail scraping
FSSC 2-d	B8603	6/18/2007	Clinical	HP08, blood
FSSC 2-d	B8604	6/18/2007	Clinical	HP08, blood
FSSC 2-d	B8605	6/18/2007	Clinical	HP08, blood
FSSC 2-d	B8606	6/18/2007	Clinical	HP08, skin biopsy
FSSC 2-d	B8607	6/19/2007	Clinical	HP08, skin swab
FSSC 2-d	B8608	6/21/2007	Clinical	HP08, sputum
FSSC 2-d	B8609	7/4/2007	Clinical	HP09, skin biopsy
FSSC 2-d	B8683	7/10/2009	Clinical	DP57, skin scraping
FSSC 2-d	B8610	7/17/2007	Clinical	HP09, blood
FSSC 2-d	B8611	7/17/2007	Clinical	HP09, blood
FSSC 2-d	B8612	7/17/2007	Clinical	HP09, blood
FSSC 2-d	B8613	7/4/2007	Clinical	HP09, skin swab
FSSC 2-d	B8616	7/26/2007	Clinical	HP10, skin scraping
FSSC 2-d	B8617	7/26/2007	Clinical	HP10, skin scraping
FSSC 2-d	B8625	8/2/2007	Clinical	HP11, nail scraping
FSSC 2-d	B8626	8/7/2007	Clinical	HP11, lesion swab
FSSC 2-d	B8649	8/10/2007	Clinical	DP22, nail scraping
FSSC 2-d	B8650	8/17/2007	Clinical	DP27, skin scraping
FSSC 2-d	B8655	9/21/2007	Clinical	DP32, skin scraping
FSSC 2-d	B8661	10/31/2007	Clinical	DP35, skin scraping
FSSC 2-d	B8749	10/31/2007	Environmental	BR07, water
FSSC 2-d	B8750	10/31/2007	Environmental	BR07, water
FSSC 2-d	B8663	1/11/2008	Clinical	DP37, nail scraping
FSSC 2-d	B8668	4/8/2008	Clinical	DP42, nail scraping
FSSC 2-d	B8669	4/24/2008	Clinical	DP43, skin scraping
FSSC 2-d	B8673	9/26/2008	Clinical	DP47, nail scraping
FSSC 2-d	B8675	10/16/2008	Clinical	DP49, nail scraping
FSSC 2-d	B8631	12/1/2008	Clinical	HP14, lesion aspirate
FSSC 2-d	B8632	12/2/2008	Clinical	HP14, skin biopsy
FSSC 2-d	B8679	2/20/2009	Clinical	DP53, nail scraping
FSSC 2-f	B8587	12/14/2005	Clinical	HP01, blood
FSSC 2-f	B8588	12/14/2005	Clinical	HP01, blood
FSSC 2-f	B8692	9/21/2006	Environmental	BR10, floor swab
FSSC 2-f	B8596	12/26/2006	Clinical	HP04, lesion ulcer
FSSC 2-f	B8597	2/4/2007	Clinical	HP05, blood
FSSC 2-f	B8598	2/4/2007	Clinical	HP05, blood
FSSC 2-f	B8599	2/5/2007	Clinical	HP05, skin biopsy
FSSC 2-f	B8600	3/12/2007	Clinical	HP06, nail scraping
FSSC 2-f	B8644	6/18/2007	Clinical	DP22, nail scraping
FSSC 2-f	B8645	6/18/2007	Clinical	DP23, skin scraping
FSSC 2-f	B8615	7/26/2007	Clinical	HP10, skin swab
FSSC 2-f	B8651	9/6/2007	Clinical	DP28, nail scraping
FSSC 2-f	B8657	10/4/2007	Clinical	DP28, nail scraping
FSSC 2-f	B8666	3/7/2008	Clinical	DP40, nail scraping
FSSC 2-f	B8671	5/14/2008	Clinical	DP45, nail scraping
FSSC 2-h	B8665	1/22/2008	Clinical	DP39, nail scraping
FSSC 2-i	B8646	6/26/2007	Clinical	DP24, nail scraping
FSSC 2-i	B8653	9/21/2007	Clinical	DP30, nail scraping
FSSC 2-i	B8670	4/28/2008	Clinical	DP44, nail scraping
FSSC 2-i	B8678	1/26/2009	Clinical	DP52, nail scraping
FSSC 2-i	B8688	10/6/2009	Clinical	DP63, nail scraping
FSSC 2-i	B8687	10/8/2009	Clinical	DP62, skin scraping
FSSC 2-k	B8642	6/5/2007	Clinical	DP20, skin scraping
FSSC 2-k	B8647	7/2/2007	Clinical	DP25, nail scraping
FSSC 2-k	B8614	7/26/2007	Clinical	HP10, skin swab
FSSC 2-k	B8618	9/5/2007	Clinical	HP10, sinovial fluid
FSSC 2-k	B8619	9/5/2007	Clinical	HP10, sinovial fluid
FSSC 2-k	B8620	9/10/2007	Clinical	HP10, sinovial fluid
FSSC 2-k	B8621	9/10/2007	Clinical	HP10, sinovial fluid
FSSC 2-k	B8654	9/21/2007	Clinical	DP31, skin scraping
FSSC 2-k	B8656	9/24/2007	Clinical	DP23, nail scraping
FSSC 2-k	B8622	10/8/2007	Clinical	HP10, sinovial fluid
FSSC 2-k	B8623	10/9/2007	Clinical	HP10, sinovial fluid
FSSC 2-k	B8624	10/11/2007	Clinical	HP10, sinovial fluid
FSSC 2-t	B8594	7/19/2006	Clinical	HP03, lesion swab
FSSC 2-t	B8595	7/19/2006	Clinical	HP03, lesion swab
FSSC 2-t	B8658	10/16/2007	Clinical	DP33, skin scraping
FSSC 2-u	B8652	9/17/2007	Clinical	DP29, nail scraping
FSSC 2-u	B8674	10/13/2008	Clinical	DP48, nail scraping
FSSC 3+4 (100)	B8677	12/19/2008	Clinical	DP51, skin scraping
FSSC 3+4 (100)	B8636	1/29/2009	Clinical	HP16, corneal ulcer swab
FSSC 5 (99.5)	B8676	10/17/2008	Clinical	DP50, nail scraping
FSSC 7 (99.7)	B8634	1/5/2008	Clinical	HP15, blood
FSSC 7 (99.7)	B8635	1/5/2008	Clinical	HP15, blood
FOSC^†^ (100)	B8698	11/1/2006	Environmental	BR03, shower swab
FOSC^†^ (100)	B8660	10/26/2007	Clinical	DP34, skin scraping
FOSC^†^ (99.7)	B8667	3/27/2008	Clinical	DP41, nail scraping
FOSC^†^ (100)	B8672	8/18/2008	Clinical	DP46, nail scraping
FOSC^†^ (99.9)	B8633	12/18/2008	Clinical	HP15, skin scraping
FOSC 33 (100)	B8699	11/1/2006	Environmental	BR03, wall swab
FOSC 33 (100)	B8701	11/1/2006	Environmental	BR03, sink swab
FOSC 33 (100)	B8702	11/1/2006	Environmental	BR03, sink swab
FOSC 33 (100)	B8691	9/21/2006	Environmental	BR10, sink swab
FOSC 33 (100)	B8695	10/5/2006	Environmental	BR11, sink swab
FOSC 33 (100)	B8713	7/31/2007	Environmental	BR06, wall swab
FOSC 33 (100)	B8719	8/14/2007	Environmental	BR04, sink swab
FOSC 33 (100)	B8722	8/15/2007	Environmental	Nephrology, wall swab
FOSC 33 (100)	B8723	8/27/2007	Environmental	BR01, sink swab
FOSC 33 (100)	B8724	8/27/2007	Environmental	BR01, faucet swab
FOSC 33 (100)	B8726	8/27/2007	Environmental	BR02, sink swab
FOSC 33 (100)	B8727	8/27/2007	Environmental	BR02, water
FOSC 33 (100)	B8729	8/31/2007	Environmental	BR05, sink swab
FOSC 33 (100)	B8730	8/31/2007	Environmental	BR05, sink swab
FOSC 33 (100)	B8731	10/16/2007	Environmental	BR08, sink swab
FOSC 33 (100)	B8732	10/16/2007	Environmental	BR08, sink swab
FOSC 33 (100)	B8733	10/19/2007	Environmental	BR09, shower swab
FOSC 33 (100)	B8734	10/19/2007	Environmental	BR09, shower swab
FOSC 33 (100)	B8735	10/19/2007	Environmental	BR09, shower swab
FOSC 33 (100)	B8736	10/19/2007	Environmental	BR09, wall swab
FOSC 33 (100)	B8737	10/19/2007	Environmental	BR09, sink swab
FOSC 33 (100)	B8739	10/23/2007	Environmental	BR10, sink swab
FOSC 33 (100)	B8740	10/23/2007	Environmental	BR11, wall swab
FOSC 33 (100)	B8629	10/24/2008	Clinical	HP13, blood
FOSC 33 (100)	B8742	10/25/2007	Environmental	BR12, sink swab
FOSC 33 (100)	B8743	10/25/2007	Environmental	BR12, shower swab
FOSC 33 (100)	B8746	10/26/2007	Environmental	BR01, water
FOSC 33 (100)	B8747	10/26/2007	Environmental	BR02, water
FOSC 33 (100)	B8748	10/26/2007	Environmental	BR02, water
FOSC 33 (100)	B8630	10/30/2008	Clinical	HP13, catheter tip
FOSC 33 (100)	B8752	10/31/2007	Environmental	BR07, water
FOSC 33 (100)	B8756	7/6/2009	Environmental	BR06, sink swab
FOSC 33 (100)	B8758	7/6/2009	Environmental	BR03, sink swab
FOSC 33 (100)	B8762	7/27/2009	Environmental	BR06, sink swab
FOSC 183 (100)	B8751	10/31/2007	Environmental	BR02, water
FIESC^†^ (98)	B8709	8/7/2007	Environmental	BR04, air
FIESC 15 (100)	B8689	6/30/2005	Environmental	BR06, air
FIESC 15 (100)	B8703	1/29/2007	Environmental	ICU, air (control)
FIESC 15 (100)	B8704	1/29/2007	Environmental	ICU, air (control)
FIESC 15 (100)	B8705	6/13/2007	Environmental	ICU, air (control)
FIESC 15 (99.5)	B8706	6/13/2007	Environmental	ICU, air (control)
FIESC 15 (100)	B8753	4/30/2009	Environmental	ICU, air (control)
FIESC 15 (100)	B8755	4/30/2009	Environmental	ICU, air (control)
FIESC 17 (100)	B8708	6/18/2007	Environmental	Pediatrics, air (control)
FIESC 20 (100)	B8707	6/18/2007	Environmental	Pediatrics, air (control)
FIESC 20 (100)	B8759	7/6/2009	Environmental	BR03, air
GFSC (94.1)	B8627	4/18/2008	Clinical	HP12, blood
GFSC (94.1)	B8628	4/18/2008	Clinical	HP12, blood

^†^ Species identity not determined due to multiple genetic matches.

Note: Each isolate was given a unique isolate number. Isolate sources have designations as follows: BR (bathroom), HP (hematology patient), DP (dermatology patient), followed by a source number. In many cases, more than one isolate was collected from the same source.

Abbreviations: FSSC, Fusarium solani species complex;FOSC, *Fusarium oxysporum* species complex; FIESC, *Fusarium incarnatum equiseti* species complex; GFSC, *Gibberella fujikuroi* species complex.

FSSC 2-d and FSSC 2-f were the most common STs isolated from patients (Figure 2). FSSC 2-d was collected from seven patients in the BTMU (20 isolates) and 12 dermatology outpatients, while FSSC 2-f was isolated from five BTMU patients (8 isolates) and five dermatology outpatients (6 isolates). FSSC 2 isolates were rarely found in the environment (*n* = 4); two FSSC 2-d isolates were collected from the same water source (BR07) on the same day, one FSSC 2-d was isolated from an air sample (BR03), and FSSC 2-f was collected from a floor swab of BR10. None of the environmental FSSC 2 isolates were collected within the same month as those collected from patients in the BMTU (Table 2).

FSSC 2-k (1 patient, 8 isolates) and FSSC 2-t (1 patient, 2 isolates) were the other STs identified by MLST in the BMTU, but none were collected in the environment (Figure 2). Other *Fusarium* isolated in hematology patients were provisionally identified by *EF-1α* sequence comparison and each was found in one BMTU patient. These were FSSC 3+4 (HP16, corneal swab), FSSC 7 (HP15, blood), FOSC 33 (HP13, blood and catheter tip), GFSC (HP12, blood), and unspeciated FSSC (HP01, blood) and FOSC (HP15, skin) (Table 2).

Among environmental isolates, FOSC 33 comprised almost half of all samples collected, and was isolated 32 times between September 2006 and July 2009, with the majority (24) sampled between August and October 2007. This species was found in water-related surface swabs of all 12 BMTU bathrooms, a nephrology wall swab (control), and water of two bathrooms (BR02, BR07). FOSC 33 was also isolated from blood and a catheter tip of a patient (HP13) in October 2008, but none were collected from the environment that month (Table 2).

Forty-three bathroom swabs were performed between September 2006 and July 2009, and 6 different *Fusarium* species/STs were recovered; FOSC (unknown species) and FOSC 33, FSSC 1-a, 1-b, 2-d, and 2-f. Water samples positive for *Fusarium* (*n* = 10) were collected in four bathrooms (BR01, 02, 03, 07) and yielded four STs; FOSC 183, FOSC 33, FSSC 1-b and FSSC 2-d. Only BR01 and BR07 were positive for FSSC (1-b, and 2-d, respectively), and water in these rooms also yielded FOSC STs. FIESC was collected from air in three bathrooms (BR03, BR04, BR06) and from 8 control samples. Three other STs were found in air, FSSC 1-a, 1-b, and 2-d, and all were collected from BR03.

## Discussion

Given the severe morbidity and mortality from *Fusarium* infections, it is important to understand the sources of these organisms and determine the extent to which fusaria may be transmitted in the hospital. One such study by Anaissie in 2001 implicated hospital water systems (drains, faucet aerators, shower heads) as potential reservoirs for *Fusarium*, and suggested that these fungi can be transmitted through the water system due to their persistence in plumbing systems [13]. In the United States, Short *et al*. recently confirmed these findings by performing an extensive MLST study of fusaria recovered from plumbing drains in the United States [14]. They found that the 6 most common sequence types in drains (FSSC 1-a, 1-b, 2-d, 2-k, FOSC 33 and *Fusarium dimerum*) were also the six most commonly associated with human infection. Our study bolsters the findings of Short *et al*. with regard to common *Fusarium* STs isolated from water-related sources, since we also found that FOSC 33 was the most common ST isolated in drains, faucets, and tiles of showers and sinks in the hospital. Unlike Short *et al*., we found FSSC 1-a and FSSC 1-b to be more commonly found in these sources, as opposed to FSSC 2-d. Our study differs in two ways, however, since environmental sampling was conducted in Brazil, and only exterior plumbing surfaces were sampled.

Several studies have shown that MLST of three loci (*EF-1α*, *rRNA* and *RPB2*) have the highest discriminatory power in delineating FSSC using comparative sequence analyses [7,15,16]. We used this three-locus method and found that subtypes of most BTMU inpatient clinical isolates belonged to FSSC species 2 (69%, 38/55), and were primarily 2-d (recovered from 36% of patients) and 2-f (recovered from 18% of patients). In contrast, FSSC isolates comprised 36% of the environmental samples and were shown to be predominantly species type 1-a (18%) and 1-b (13%), while only four environmental samples were FSSC 2. The remaining environmental samples were predominantly FOSC from swabs and water (50% of environmental samples) and FIESC from air (79% of air samples). We did not find any FIESC clinical isolates, although we did find two FOSC clinical cases. Our data suggest that most of the clinical cases of fusariosis in this immunosuppressed population could not be traced to a specific water-related environmental source in the hospital ward. However, because we found a small number of species type-level matches between clinical and environmental isolates, we believe that in this institution, environmental water-related sources and even air must be considered as possible reservoirs for patient infection.

In this study, we were unable to find any temporal association between BMTU patient fusarial infections and contaminated water-related sources in the BMTU environment. We explored the epidemiology of community acquisition by studying a population of dermatology outpatients diagnosed with skin or nail fusariosis who were seen at the hospital during the same period. The spectrum of *Fusarium* STs recovered from these patients largely mirrored those recovered from BMTU inpatients (Figure 1). A study by Raad *et al*. used RAPD subtyping to demonstrate that *Fusarium* DNA collected from 10 infected patients did not match DNA from 15 environmental isolates collected from shower head water, sink water, and drains in their institution [17]. Our study was far more extensive and yielded similar results.

Both Anassie and Short [13,14] have implicated water systems as reservoirs of fusariosis, but in our study we found only FSSC 1 to be common in both hematology patients and the environment. It is possible that *Fusarium* patient subtypes could have been missed in the hospital environment because of limitations in the environmental investigation. Environmental sampling was sporadic and was limited in scope, so potential sources of contamination may have been missed. Few samples were collected from air in the BMTU, and surfaces of other fomites in the patient rooms were not sampled; rather focus was placed on the patient bathrooms, pursuing the hypothesis that water-related sources were *Fusarium* reservoirs. Additionally, the methods used for environmental sample collection may not have been optimal for *Fusarium* recovery.

The diversity of *Fusarium* STs among dermatology patients with superficial infections was greater than that of BMTU patients, and the distribution of STs was highly similar between these patient populations. We found that a single species, FSSC 2-d, predominated in both inpatient hematology and outpatient dermatology samples. Certainly, our combined patient data suggest that FSSC species remain a cause of superficial colonization, and under appropriate circumstances of immunosuppression, could cause disease in an IHC patient population. The increase in fusariosis in this and other hospitals (personal communication, Marcio Nucci) suggests that there may be a significant increase in circulating *Fusarium* conidia in this geographical region. Our findings raise important questions for future research, including a search for the environmental reservoirs of *Fusarium* spp., an explanation for this surge of environmental *Fusarium*, and the potential implications of these findings in infection control measures.

## Conclusions

We isolated similar *Fusarium* species in this hospital and outpatient setting. The limited environmental investigation coupled with molecular analysis did not show a definitive link to clinical isolates causing infection. Future studies are needed in order to identify reservoirs of *Fusarium* in the community, as well as preventive measures for patients at high risk for IF.

## Abbreviations

BMTU: Bone marrow transplant unit; CDC: Centers for Disease Control and Prevention; EF-1α: Elongation factor 1-*alpha*; IF: Invasive Fusariosis; FIESC: *Fusarium incarnatum-equiseti* species complex; FOSC: *Fusarium oxysporum* species complex; FSSC: *Fusarium solani* species complex; GFSC: *Gibberella fujikuroi* species complex; ITS: Internal transcribed spacer region; ML: Maximum likelihood; MLST: Multi-locus sequence typing; MP: Maximum parsimony; NJ: Neighbor joining; *RPB2*: RNA polymerase II subunit; *rRNA*: rRNA DNA region; RT: Room temperature; SDA: Sabouraud dextrose agar; SDA + C: Sabouraud dextrose agar with chloramphenicol; ST: Species type.

## Competing interests

The authors declare that they have no competing interest.

## Authors’ contributions

SAB, MN and CMS conceived the study design and prepared article draft. CMS and SFH performed PCR and molecular analysis. GB and TA performed environmental sampling, fungal isolation and cultures. MN provided patient care and management and patient isolate collection. All authors contributed to, reviewed, and approved the final draft of this article.

## Authors’ note

The findings and conclusions in this report are those of the authors and do not necessarily represent the views of the Centers for Disease Control and Prevention.

## Pre-publication history

The pre-publication history for this paper can be accessed here:

http://www.biomedcentral.com/1471-2334/13/49/prepub
